# RipetaScore: Measuring the Quality, Transparency, and Trustworthiness of a Scientific Work

**DOI:** 10.3389/frma.2021.751734

**Published:** 2022-01-21

**Authors:** Josh Q. Sumner, Cynthia Hudson Vitale, Leslie D. McIntosh

**Affiliations:** ^1^Ripeta, LLC, St. Louis, MI, United States; ^2^Washington University School of Medicine, Donald Danforth Plant Science Center, Washington University in St. Louis, St. Louis, MI, United States; ^3^Association of Research Libraries, Washington, DC, United States

**Keywords:** research metrics, research quality, scientific indicators, reproducibility, research integrity

## Abstract

A wide array of existing metrics quantifies a scientific paper's prominence or the author's prestige. Many who use these metrics make assumptions that higher citation counts or more public attention must indicate more reliable, better quality science. While current metrics offer valuable insight into scientific publications, they are an inadequate proxy for measuring the quality, transparency, and trustworthiness of published research. Three essential elements to establishing trust in a work include: trust in the paper, trust in the author, and trust in the data. To address these elements in a systematic and automated way, we propose the ripetaScore as a direct measurement of a paper's research practices, professionalism, and reproducibility. Using a sample of our current corpus of academic papers, we demonstrate the ripetaScore's efficacy in determining the quality, transparency, and trustworthiness of an academic work. In this paper, we aim to provide a metric to evaluate scientific reporting quality in terms of transparency and trustworthiness of the research, professionalism, and reproducibility.

## Introduction

Misinformation, disinformation, and a general distrust in research and science by members of the general public has been the topic of many news stories in the last few years. This has cascaded into a series of funding policies, executive memos, and national and international task forces being established to increase research integrity and restore trust in scientific outcomes and policies that have resulted from those outcomes (United States White House, 2021). One critical factor in enhancing trust in research is through the increased transparency of reporting research (Moher et al., 2020). Yet, few tools and even fewer assessment metrics exist to evaluate the responsible reporting of research.

Existing research assessment metrics purport to measure a paper's quality or an author's clout. Often the fields of quality and prominence are lumped together, with authors that consistently have high citation counts being assumed to conduct the best research. Despite this conflation of quantity and prestige, there is plenty to be gained in various parts of the research world from metrics such as the H-index (Hirsch, 2005), RG score (ResearchGate, 2021), and Altmetric (Digital Science, 2018a) which all provide valuable insight for certain applications. Still, none of these measures serve as an appraisal of how trustworthy or reproducible a publication is based on the paper's content. Instead, these measures tend to track popularity or impact using publicly available information about the spread and influence of a paper or an author. While these are useful quantities, they should not be treated as direct measures of credibility, rigor, or quality of a publication. In light of the publishing frenzy and heightened media attention on research through the COVID-19 pandemic there is a growing need for a user-oriented guide to understand the quality of a specific scientific paper. We propose our novel ripetaScore to address this need and serve as a direct measurement of the quality of a paper. In this paper, we aim to introduce this metric to evaluate scientific reporting quality in terms of transparency and trustworthiness through use of the three-part ripetaScore, measuring research, professionalism, and reproducibility.

The trust in reproducibility score is centered around the elements of a paper, which may facilitate a future researcher to most accurately replicate the study. Ripeta is a technology company that has developed tools and services to automatically assess the responsible reporting of research. Ripeta tools extract and show the responsible reporting of key scientific quality indicators within a scientific paper. Ripeta scans the paper's text for a selection of indicators spanning methodology, availability of data or code, analysis process, and analysis software citation. Though reproducibility is not guaranteed, if these variables are present in a paper then there is higher potential for reproducibility and are clear scientific quality indicators. Reproducibility is important for using research funds appropriately, providing the most reliable information possible, and for strengthening ethical scientific practices.

The trust in professionalism score aims to measure the legitimacy of the paper's authors and their thoroughness in reporting outside influences on their work. Two pieces currently play in determining the trust in professionalism: (1) Identifying if an author is who they say they are; and (2) Determining whether or not the authors are adhering to reporting standards put in place, such as being open about ethical declarations, conflicts of interest, and funding sources.

Finally, the trust in research component determines whether a paper meets general specifications for what “research” is. While this is normally obvious when reading a manuscript, it is important to factor into any analyses using automated methods. Some publishers do not make obvious distinctions in paper metadata between editorials or communications and research articles. For that reason, our score contains a “Trust in Research” component.

## Materials and Methods

The goal for the ripetaScore is to provide a meaningful high-level score summarizing the process of verifying the quality reporting of research and manuscript structure so experts can then more easily check the science. While other tools evaluate some scientific reporting practices, none are implemented as a complete summary of a publication like the ripetaScore. For the ripetaScore, we leveraged a locally developed corpus of publications to create the training dataset as well as personal experiences evaluating papers through the research lifecycle. The corpus of papers was selected based on several criteria and in response to internal needs for more data and external requests for analysis. Broadly, the training dataset was comprised of publications:

- Searchable by DOI
- Published either through peer-reviewed journal or hosted on a preprint server
- Recorded in the Dimensions database (Digital Science, 2018b)
- Licensed CC-BY or CC-0 or with access allowed via contractual permission.

Papers meeting the criteria above were collected and their text stored for use by leveraging various natural language processing (NLP) models. Ripeta has developed several NLP models, each tuned to a specific scientific quality indicator and based upon previous research conducted in developing the Ripeta reproducibility framework (McIntosh et al., 2017). Trained to read like humans, these NLP models scan articles for seed phrases and terms that indicate the presence of their respective quality indicator. These models were developed iteratively through a workflow utilizing human annotations and machine learning algorithms. The first stage of development involved manually annotating scientific papers for such aspects as data availability statements, explanations of statistical procedures and software, or study purposes to provide seed terms and known true positive results for a set of publications. These annotations were carried out in prodigy (Prodigy, 2021) for convenient integration with Ripeta's corpus of papers and with SpaCy (Honnibal and Montani, 2017). Next, a SpaCy model was built that used the extracted terms and examples to look for linguistic patterns to find similar phrases in the manually annotated papers as well as new publications. These steps were repeated many times until our NLP models could reliably return accurate results for new publications without any human guidance. Besides yielding very precise models this process has ensured that as the scientific landscape develops, Ripeta can retrain these NLP models to react to emerging challenges or to uphold more stringent standards. Now able to process the manuscripts, the NLP models extract text they recognize as matches for their respective criteria, based on those criteria's definitions.

From our total corpus of over a half a million articles, a subset of 12,000 CC-BY and CC-0 publications was selected to develop and test the ripetaScore. That sample included a variety of subject areas, funders, publishers, and journals. Publications that were not research articles were excluded as part of the scoring process.

**Figure 1:**
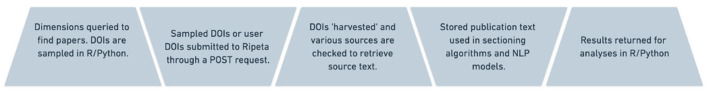
Manuscript collection workflow.

As shown in Figure 1, once papers were identified, a persistent identifier such as a DOI or PMCID was submitted for each publication that should be collected by Ripeta via a POST request. We then validated the ID format and searched the Dimensions database for the identifier. If the publication exists in Dimensions, the DOI and other paper metadata were collected and a unique identifier to be used internally was assigned to the paper. CrossRef (2021) and Unpaywall (2021) were checked for additional paper metadata and license information is checked against Ripeta policy. If the license information did not meet policy, the paper was not stored in Ripeta's corpus and the harvesting process was terminated. Otherwise, the source document URLs were collected then the source document was parsed and stored for later use. Harvested papers were cleaned and sectioned using the papers' XML to allow for algorithm development.

Next, papers were run through appropriate NLP models. These NLP models were created using Python version 3.7 (Van Rossum, 2007), SpaCy version 2.3.5 (spaCy, 2021), and Prodigy version 1.10.3 (Prodigy, 2021) at time of writing. The models have been trained to read similarly to how humans do. Model training started with a selection of seed terms, keywords, or phrases expected to be in a given statement, and proceeded from there to find more complex patterns among human-annotated training and testing papers. Seed terms and other model parameters were iterated upon until our desired accuracy was achieved, thereafter performance was measured continuously to ensure high accuracy. Results from the various NLP models were analyzed in R version 4.0.2 and/or Python version 3.7 to create reports, interactive dashboards, or other data summaries.

**Figure 2:**
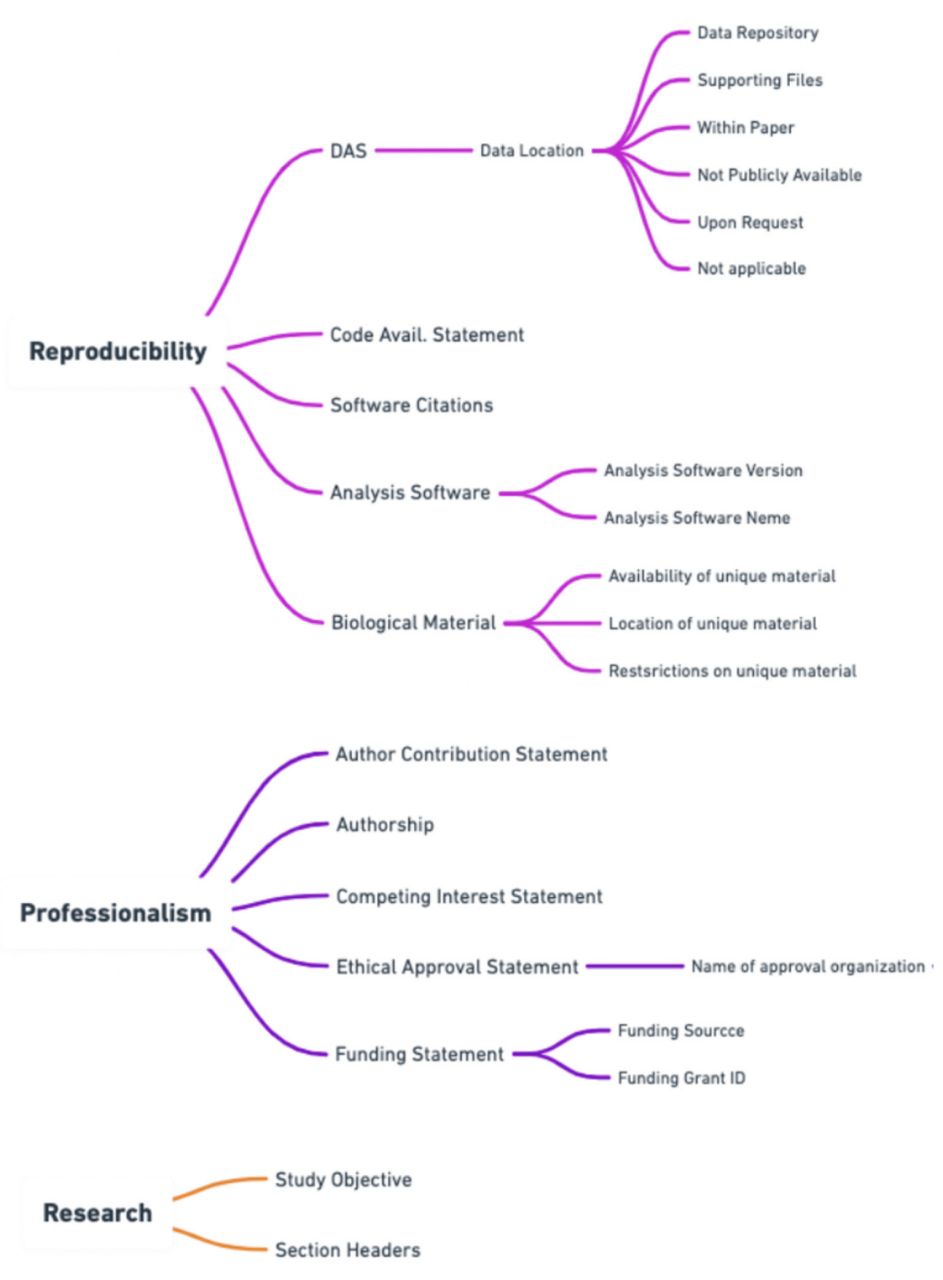
Additional subsections within the ripetaScore that provide a more granular representation of the scientific quality of the manuscript.

To best capture the trustworthiness of a publication the score needed both a professionalism and reproducibility component. Please see Figure 2 for a breakdown of how scientific quality indicators were categorized into areas of research, professionalism, and reproducibility by Ripeta. Many of the scientific quality indicators were further subsectioned into more granular components based on the returned text response from the manuscript.

## The RipetaScore Scoring Workflow

### Components of the RipetaScore: Research, Professionalism, and Reproducibility

**Figure 3:**
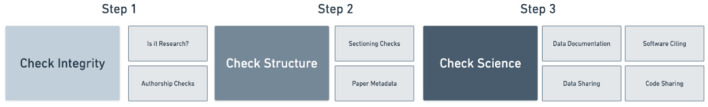
RipetaScore scoring workflow.

The ripetaScore combines three aspects of trust for a total of 30 points. See Figure 3 for the ripetaScore scoring workflow. First, a paper is analyzed across our “Trust in Research” criteria to determine whether the paper is a research paper, which determines whether the paper will continue to be scored or not. Research articles are then evaluated for the presence of our reproducibility quality indicators and receive up to 20 points from those criteria. The last 10 points come from our trust in professionalism quality indicators.

The first step in scoring a manuscript is determining whether the document is a research paper or not. There are a variety of things that are not considered “real research” for one reason or another. An example criteria is that scientific research should have certain enumerated divisions separating the manuscript into recognizable sections. If key sectioning is not present, such as methods or conclusions, that would contribute to an indication that an article may not be scientific research. Additionally, the content of some work may flag it as something other than research. Titles and language outside of the normal scientific lexicon may simply be authors expressing themselves in their work, but in some cases it can be a useful tool in evaluating a paper. To make science better the community needs to be able to quickly and effectively determine what is research and what is not. For the purposes of scientific betterment, it does not matter if the statistics are reported well, if the publication was churned out of a paper mill. For such a publication, arguing minutiae of the methods misses the real issue and frames the discussion in an unproductive manner. With the goal of scientific betterment in mind, papers in our corpus were evaluated by our NLP algorithms and selections of papers were manually reviewed to examine correlations between quality of publications and different quality indicators Ripeta has developed. Removing these non-research articles from the corpus of scored papers increases Ripeta's efficiency through the rest of the scoring process and clearly differentiates research that is lacking in key quality indicators from submissions that simply are not research articles.

Papers determined to be real research are evaluated to gauge the trust in their reproducibility. Trust in reproducibility encompasses the majority of the ripetaScore for research articles. Since the widespread acknowledgment of a reproducibility crisis in science there has been much discussion about how to remedy these problems (Strech et al., 2020). The trust in reproducibility component of the ripetaScore is based on quality indicators designed to address this crisis. Primarily papers are evaluated with regards to their data/code sharing practices, thoroughness in explaining methods, and citing software. These indicators were picked due to their role in improving the likelihood of a study being well-enough documented as to be fully reproducible (Vickers, 2006; Baggerly, 2010). While these indicators are important, they cannot guarantee that a published finding is correct and fully reproducible. For example, currently we identifies whether data was shared and if so where the data was shared, but we are not yet making efforts to retrieve the data to assess its quality. Similarly, we look for evidence that the methods are sufficient to describe the work in detail, but we do not assess the methods for their appropriateness to a given field or study design. As we continue to develop new algorithms this component of the score may grow in scope.

Finally, papers are evaluated for trust in professionalism. Trust in professionalism is about components of research such as authorship and scientific etiquette. Some of the main contributors to this trust in professionalism aspect of the ripetaScore include whether ethical approval is properly cited, how corresponding authors can be reached, and whether funding sources are disclosed. Authorship concerns are another factor being incorporated into trust in professionalism. Over the past decade there have been nearly 2,800 retractions due to authorship issues (Retraction Watch Database, 2021). The reasons for these retractions range from forged authorship and faked peer review to uncovered paper mills or author misconduct. Retractions are not only harmful to a journal's reputation, they also waste a tremendous amount of resources and can lead to negative repercussions in public policy as seen with several COVID-19 publications and preprints (Stern et al., 2014; Davido et al., 2020; Retracted COVID-19 papers, 2021). We used retrospective analysis of these retractions as well as exploratory analysis of preprints as they were submitted during the COVID-19 pandemic to develop a list of use cases for authorship trust, or trust in professionalism. Mainly we are interested in reducing the burden to journals and publishers by separating manuscripts where the authorship requires manual review from those manuscripts where professionalism can be established using existing data sources and the content of the paper. Through developing these use cases, collecting paper metadata, and analyzing the content of an academic paper our scoring criteria provide a useful metric for detecting potentially worrisome authorship issues. The journal putting forth an article also plays an important role in professionalism. Namely, suspected predatory journals and publishers should be monitored and their influence needs to appear in any evaluation of scientific trust. Finally, there are important parts of scientific etiquette that are evaluated as part of trust in professionalism. Widely accepted best practices such as stating funding sources or listing ethical approvals are important to the integrity of research and to professional scientific conduct. These best practices along with authorship checks inform our trust in professionalism score. In aggregate, trust in professionalism reflects on journal practices but for a single paper this reflects on individual trustworthiness of the work.

Together these three components of the ripetaScore make for an automated, holistic evaluation of the quality of a scientific text, which is useful to everyone from casual readers to journal editors looking to save time and money during costly procedures.

## Results

### Evaluating the RipetaScore

There are many components of scientific quality and all of them should be taken into account when evaluating a publication. To help make these concepts more concrete we will go over a few examples of papers that score well or that score poorly using our ripetaScore. In order to avoid drawing unwanted attention to individual authors these papers are presented anonymously but with some context surrounding the field of research, journal, or time of publication.

The calculation for the ripetaScore is weighted across Ripeta's scientific quality indicators. The first component, the check for “Research” is based on whether or not the article is a true research paper or not. This is calculated as a simple pass (1) or fail (0) in the score creation. The next components of the ripetaScore— “Professionalism” and “Reproducibility” —assess how the paper performs across quality indicators. Each “Professionalism” quality indicator is assigned a numerical representation based on its importance for responsible reporting practices with a maximum score of 10. Finally, a “Reproducibility” score is calculated based on the numerical representation assigned for each quality indicator supporting the potential to reproduce the work with a maximum score of 20. The calculation is: ripetaScore = Research Check (pass/fail) ^*^ [Professionalism (0–10) + Reproducibility (0–20)].

The first example (Table 1) paper scores quite well with a ripetaScore of 23. This paper was published in 2019 in PLoS Computational Biology, well after open access practices have become commonplace and in a journal known for high standards of transparency. This paper's score reflects that it includes a clear study purpose, states the funding sources and their roles, and has an ethical statement (although the ethical statement does not list specific IRB approval). Looking at the reproducibility focused criteria this paper scores nearly a perfect 20. Data and code, which overlap for this particular paper, are on Github with links provided, both of which factor heavily into the ripetaScore's reproducibility component. One place where this paper could score higher is in properly referencing MATLAB software that was used for analyses. Additionally, every author on the paper is on ORCID, although they do not all have their ORCID profiles listed in the paper's authorship information suggesting that they may not have been on ORCID when the paper was originally published. There is no evidence of any authorship issues regarding this paper. A high score such as this one is a code of confidence but would carry slightly different connotations depending on how the ripetaScore is implemented. On a preprint server it may serve to expedite the publication process and to provide readers of preprints with some baseline information about the writing. For a journal editor reviewing publications for acceptance the ripetaScore provides a quick way of assessing general quality which would both aide in most efficient use of expensive reviewer time and help serve as a check on journal policy compliance (such as whether or not submissions are adhering to a data sharing policy that is in place).

**Table 1:** RipetaScore breakdown across scientific quality indicators.

	**Ripeta's scientific quality indicators**			
	**Research check (pass/fail)**	**Professionalism (0–10)**	**Reproducibility (0–20)**	**Total score**
• **Perfect paper** • All indicators available	Pass	10	20	30
• **Research paper 1** • Missing a few indicators but overall robust documentation and potential for reproducibility	Pass	6	17	23
• **Research paper 2** • Includes only a minimum number of indicators such as an ethics statement and software used for analyses	Pass	3	7	10
• **Commentary** • Does not meet the requirements to be considered research	Fail	–	–	–

Our next example (Table 1 “Research Paper 2”) comes from a paper that scores in the lower middle of the distribution with a ripetaScore of 10 but which could score much higher with a few small improvements. This article was published in 2019 by Nature in the Scientific Reports journal. The authors of paper #2 fulfilled many of the components that go into the ripetaScore but several key indicators are still missing. For example, the paper does contain a data availability statement, but the data are listed as being available upon request from the authors, a method for data sharing that is dubiously helpful at best with under 20% of such statements enabling data to be found in many cases (Vines et al., 2013). While the authors make mention of using Graphpad Prism there is no citation to the specific version or way of examining any analysis code, both of which negatively impact the reproducibility of the work. Due to these and other factors this paper scores in the lower middle section of our possible distribution, along with the majority of other papers. A paper scoring in this range could indicate to preprint readers that they may want to pay extra attention to details about the analyses or check the author's publishing history depending on how the work did on each aspect of the ripetaScore. For a journal editor this type of score may suggest that the standard review process will be sufficient but that the paper should not be put on a fast-track to publication based on quality alone. As an author, a ripetaScore in this range is a sign that your manuscript may be lacking in some key factors aiding reproducibility, many of which are easy to add in and greatly improve the spirit of open science.

Finally, some writings such as our last example, measure up extremely poorly using the ripetaScore with a score of zero. This article was published in late 2020 by Openventio in the “Epidemiology—Open Journal.” This publication contains none of the key quality indicators that currently factor into the ripetaScore and it raises questions of authorship trustworthiness. While this combination of missing information and abnormal authorship could be evidence of a new researcher unfamiliar with best practices it may also be evidence of predatory exploitation of open science, particularly considering the subject matter (Heimstadt, 2020). When dealing with a socially or politically charged subject matter it is also important to bear in mind that the ripetaScore does not take conclusions or press attention into account. Thus, good science can be conducted then co-opted by any number of agenda's without that misuse being tracked in the ripetaScore. As a reader either of preprints or published literature, a very low ripetaScore or outlandish media claims should or a score of zero should lead to careful consideration of the claims and whether they make sense given the rest of the scientific literature. From the perspective of a journal editor this sort of ripetaScore should raise red flags and suggest that extensive review and revisions may be necessary to get the paper to meet a journal's quality standards. Lastly, for an author this ripetaScore provides feedback that there are some very important components of scientific literature that are missing in your work and that the discrepancy should be addressed. In such situations we encourage authors to reference journal policies and consider existing tools meant to aid in transparency and reproducibility. There are a variety of existing options to make sharing data, protocols, and code easier such as gigantum, github, protocols.io, codeocean, figshare, and jupyter. By implementing these existing options a low ripetaScore can often be greatly improved with relatively little added effort.

**Figure 4:**
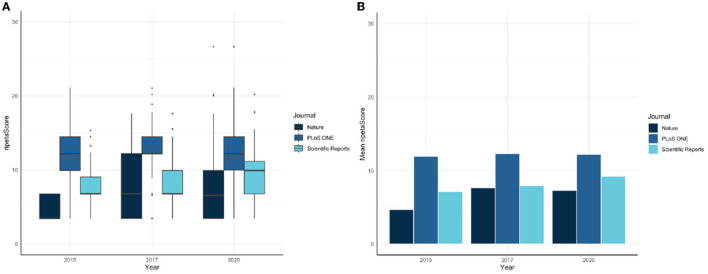
**(A,B)** RipetaScore comparison across Nature, PloS One, and Scientific Reports.

The ripetaScore is most useful when aggregated across time for a scholarly entity. As an example, Figure 4 illustrates a comparison across Nature, PloS ONE, and Scientific Reports with the average ripetaScore of publications from 2015, 2017, and 2020 in those journals.

**Figure 5:**
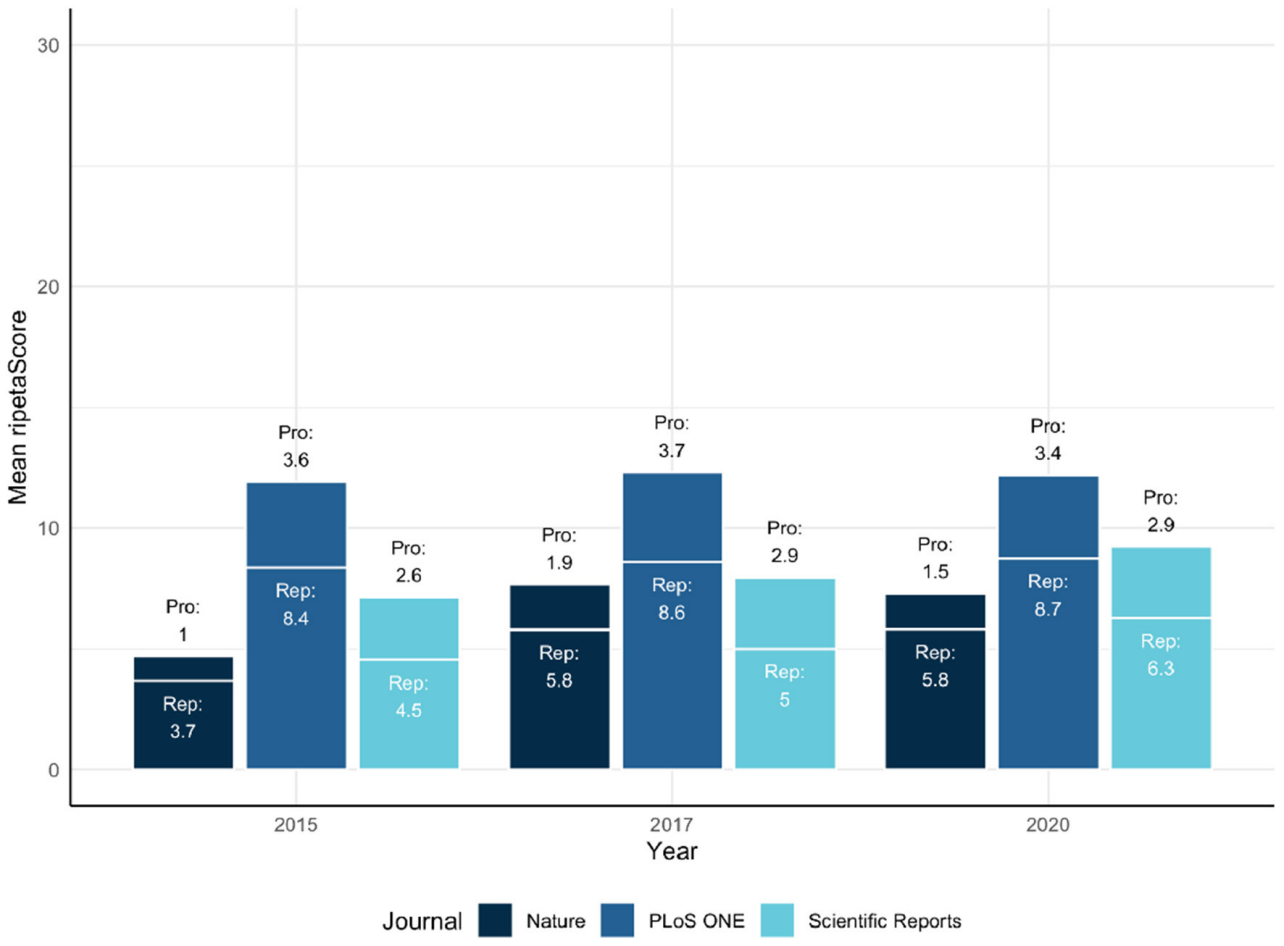
Mean ripetaScore for Nature, Plos One, and Scientific Reports.

In these comparisons it is clear that PLoS ONE is leading the other journals on average, but has relatively constant ripetaScores over time (Figure 5). Nature and Scientific Reports on the other hand have lower averages but have shown improvement over time. Looking at the score as it's component pieces it becomes clear that most of the improvements being seen are coming from reproducibility practices improving while professionalism has stayed relatively similar in most cases.

### Next Steps for the RipetaScore

While making the ripetaScore we realized that authorship was a critical component of scientific trust which could not be evaluated using our other textual variables aimed at reproducibility. Once we decided to explicitly include score aspects aimed at trustworthy authorship we investigated possible avenues of examining authorship such as network analysis and name disambiguation. As our authorship identification and evaluation processes become more refined the ripetaScore will only become more accurate and more helpful in establishing authorship trust. Similarly, as expectations or best practices for research reproducibility continue to develop, our ripetaScore will respond to those changes and to the size of our paper corpus growing.

## Conclusion

Transparency, reproducibility, and responsible scientific practices are of utmost importance to the furtherance of research and scientific betterment. The ripetaScore provides an easily accessible metric to evaluate scientific reporting quality and trustworthiness toward evaluating these ends. The ripetaScore comprises three parts, trust in research, in reproducibility, and in professionalism. These categories and their contributions to the total ripetaScore have been developed through extensive testing of Ripeta's growing corpus of scientific papers. The ripetaScore is useful in evaluating single papers or conglomerated research and with continuing development of new NLP models, new standards for reproducibility, and integration of more authorship checks the ripetaScore will only show more insights into research papers.

## Limitations

All research metrics have limitations on their applicability and use. Additionally, for all metrics the inputs to conducting these calculations change over time and as more research is published. The ripetaScore has these same limitations. While we have built a score that is extensible as the number of scientific quality indicators increases, any metric should not be the sole basis for evaluation and assessment. Rather, any of this metric should be used in context with additional evaluative techniques.

## Data Availability Statement

The datasets presented in this study can be found in online repositories. The names of the repository/repositories and accession number(s) can be found below: https://ripeta.figshare.com/.

## Author Contributions

LM and CV contributed to conception and design of the study. JS analyzed the data with input from LM. JS drafted the manuscript with editing from LM and CV. All authors contributed to manuscript revision, read, and approved the submitted version.

## Conflict of Interest

JS, CV, and LM advise or work for Ripeta as employees.

## Publisher's Note

All claims expressed in this article are solely those of the authors and do not necessarily represent those of their affiliated organizations, or those of the publisher, the editors and the reviewers. Any product that may be evaluated in this article, or claim that may be made by its manufacturer, is not guaranteed or endorsed by the publisher.

## References

[B1] Baggerly K.. (2010). Disclose all data in publications. Nature 467, 401. 10.1038/467401b 20864982

[B2] Davido B. Lansaman L. Bessis S. Lawrence C. Alvarez J. C. Mascitti H.. (2020). Hydroxychloroquine plus azithromycin: a potential interest in reducing in-hospital morbidity due to COVID-19 pneumonia (HI-ZY-COVID)? medRxiv Preprint. 10.1101/2020.05.05.20088757

[B3] Digital Science. (2018a). Altmetric [Software]. Available online at: https://www.altmetric.com/ (accessed July 31, 2021), under licence agreement.

[B4] Digital Science. (2018b). Dimensions [Software]. Available online at: https://app.dimensions.ai (accessed July 31, 2021), under licence agreement.

[B5] Heimstadt M.. (2020). Between Fast Science and Fake News: Preprint Servers Are Political. Available online at: https://blogs.lse.ac.uk/impactofsocialsciences/2020/04/03/between-fast-science-and-fake-news-preprint-servers-are-political/ (accessed May 20, 2021).

[B6] Hirsch J. E.. (2005). An index to quantify an individual's scientific research output. Proc. Natl. Acad. Sci. U.S.A. 102, 16569–16572. 10.1073/pnas.0507655102 16275915 PMC1283832

[B7] Honnibal M. Montani I. (2017). spaCy 2: Natural *Language Understanding* with Bloom *Embeddings, Convolutional Neural Networks and Incremental Parsing*.

[B8] McIntosh L. D. Juehne A. Vitale C. R. H. Liu X. Alcoser R. Lukas J. C.. (2017). Repeat: a framework to assess empirical reproducibility in biomedical research. BMC Med. Res. Methodol. 17, 143. 10.1186/s12874-017-0377-6 28923006 PMC5604503

[B9] Moher D. Bouter L. Kleinert S. Glasziou P. Sham M. H. Barbour V.. (2020). The Hong Kong Principles for assessing researchers: fostering research integrity. PLoS Biol. 18, e3000737. 10.1371/journal.pbio.3000737 32673304 PMC7365391

[B10] Prodigy. (2021). Available online at: https://prodi.gy/ (accessed July 31, 2021).

[B11] ResearchGate. (2021). Research Gate Score. Available online at: https://www.researchgate.net/ (accessed July 31, 2021).

[B12] Retracted COVID-19 papers. (2021). Retracted Coronavirus COVID-19 Papers. Available online at: https://retractionwatch.com/retracted-coronavirus-covid-19-papers/ (accessed July 31, 2021).

[B13] Retraction Watch Database. (2021). Available online at: http://retractiondatabase.org (accessed July 31, 2021).

[B14] spaCy. (2021). Available online at: https://spacy.io/ (accessed July 31, 2021).

[B15] Stern A. M. Casadevall A. Steen R. G. Fang F. C. (2014). Financial costs and personal consequences of research misconduct resulting in retracted publications. eLife 3, e02956. 10.7554/eLife.02956 25124673 PMC4132287

[B16] Strech D. Weissgerber T. Dirnagl U. QUEST Group. (2020). Improving the trustworthiness, usefulness, and ethics of biomedical research through an innovative and comprehensive institutional initiative. PLoS Biol. 18, e3000576. 10.1371/journal.pbio.3000576 32045410 PMC7012388

[B17] United States White House. (2021). Memorandum on Restoring Trust in Government Through Scientific Integrity and Evidence-Based Policymaking. Available online at: https://www.whitehouse.gov/briefing-room/presidential-actions/2021/01/27/memorandum-on-restoring-trust-in-government-through-scientific-integrity-and-evidence-based-policymaking/ (accessed September 9, 2021).

[B18] Van Rossum G.. (2007). Python *Programming Language*.

[B19] Vickers A. J.. (2006). Whose data set is it anyway? Sharing raw data from randomized trials. Trials 7, 15. 10.1186/1745-6215-7-15 16704733 PMC1489946

[B20] Vines T. Albert A. Y. K. Andrew R. L. Débarre F. Bock D. G. Franklin M. T.. (2013). The availability of research data declines rapidly with article age. Curr. Biol. 24, 94–97. 10.1016/j.cub.2013.11.014 24361065

